# Supplementary material for: Comparative meta-analysis of barely transcriptome: Pathogen type determines host preference
Source: PLoS One. 2025 Jun 30;20(6):e0320708. doi: 10.1371/journal.pone.0320708 (PMC12208424; doi:10.1371/journal.pone.0320708)
Supplement: S1 Fig — (a) The plot box of the E-GEOD-20279 dataset related to pathogens stresses in the Affymetrix platform with 3 control samples and 3 treatment samples, was drawn in the pre-normalization stage. (b) The plot box of the E-GEOD-20279 dataset after normalization, where all comparisons that are not significant or are not equal to the change threshold are converted to a log 2 value to remove a possible error. This method ensured that weak expression fluctuations were more likely to be real biological signals than measurement errors or errors not corrected by RMA normalization. The biological errors and batch effects have been corrected. After preprocessing, the black lines of the box plot are almost on the same straight line, indicating a high level of normalization. The horizontal axis stands for the control and treatment of different samples, while the vertical axis represents the expression value. The black line in the box represents the expression median for each sample. (DOCX) [file pone.0320708.s001.docx]

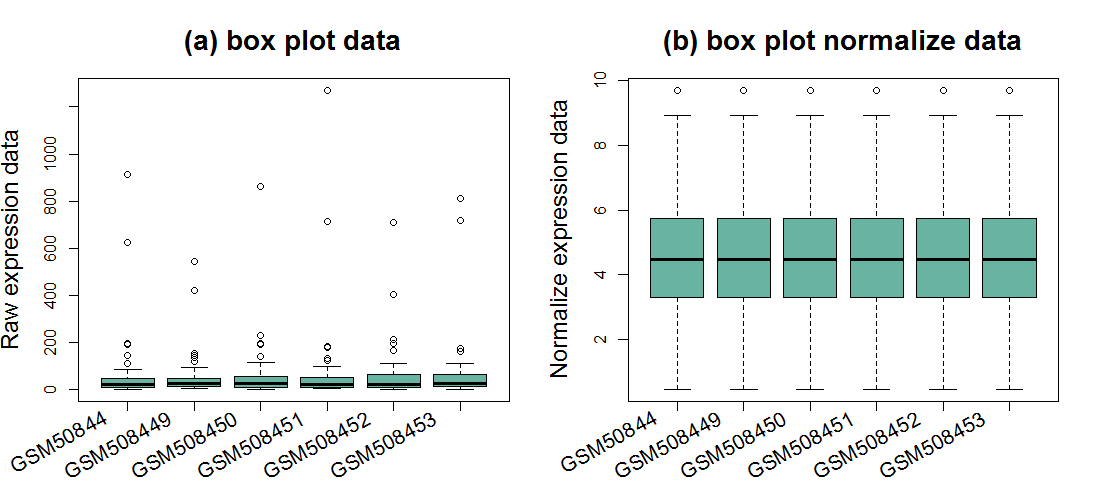


**S1 Fig.** Reducing heterogeneity among samples of studies for direct merging meta-analysis. (a) The plot box of E-GEOD-20279 dataset related to pathogens stresses in Affymetrix platform with 3 control samples and 3 treatment samples, was drawn in the pre-normalization stage. (b) The plot box of E-GEOD-20279 dataset after normalization, where all comparisons that are not significant or are not equal to the change threshold are converted to a log 2 value to remove a possible error. This method ensured that weak expression fluctuations were more likely to be real biological signals than measurement errors or errors not corrected by RMA normalization. The biological errors and batch effects have been corrected. After preprocessing, the black lines of box plot are almost on the same straight line, indicating a high level of normalization. The horizontal axis stands for control and treatment different samples, while the vertical axis represents expression value. The black line in the box represents the expression median for each sample.
