# Supplementary material for: Comparative meta-analysis of barely transcriptome: Pathogen type determines host preference
Source: PLoS One. 2025 Jun 30;20(6):e0320708. doi: 10.1371/journal.pone.0320708 (PMC12208424; doi:10.1371/journal.pone.0320708)
Supplement: S10 Table — (DOCX) [file pone.0320708.s011.docx]

**Table S10. a) The conserved cis-acting elements found in promoter of hormonal DEGs by the MEME analysis.**

| **Significant GO term identified by GOMO** | **Best match in JASPAR and PLACE** | **Width** | ***E-value*** | **Motif Logo** | **Motif name** |
| --- | --- | --- | --- | --- | --- |
| MF: TF activity CC: nucleus, plasma membrane BP: regulation of transcription, DNA-dependent | MA1267.1 | 21 | 5.7e-101 | 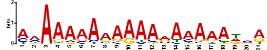 | Motif 1 |
| MF: TF activity CC: nucleus, plasma membrane | MA0150.2 | 50 | 1.6e-074 | 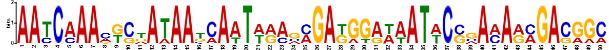 | Motif 2 |
| MF: TF activity, protein serine/threonine kinase activity CC: nucleus, plasma membrane BP: regulation of transcription, DNA-dependent | MA0072.1 | 50 | 8.2e-071 | 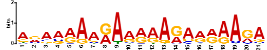 | Motif 3 |
| CC: chloroplast BP: regulation of transcription MF: TF activity | MA1268.1 | 50 | 3.0e-069 | 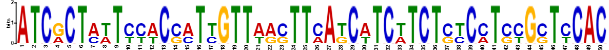 | Motif 4 |
| CC: chloroplast | MA1277.1 | 50 | 2.1e-068 | 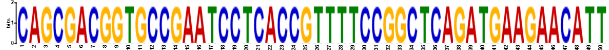 | Motif 5 |
|  | MA1260.1 | 50 | 6.4e-058 | 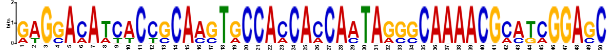 | Motif 6 |
|  | MA1267.1 | 21 | 9.0e-058 | 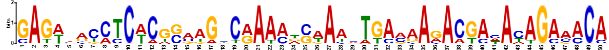 | Motif 7 |
|  | MA0777.1 | 50 | 3.1e-054 | 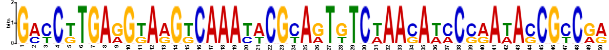 | Motif 8 |
| MF: TF activity | MA0680.1 | 50 | 4.5e-054 | 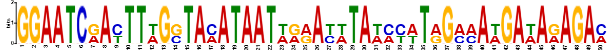 | Motif 9 |
|  | MA0578.1 | 50 | 1.2e-053 | 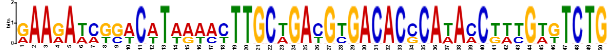 | Motif 10 |
|  | MA0415.1 | 50 | 9.0e-053 | 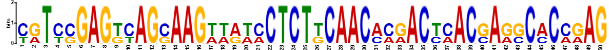 | Motif 11 |

**Table S10. b) The conserved cis-acting elements found in promoter of fungal DEGs by the MEME analysis.**

| **Significant GO term identified by GOMO** | **Best match in JASPAR and PLACE** | **Width** | ***E-value*** | **Motif Logo** | **Motif name** |
| --- | --- | --- | --- | --- | --- |
| MF: TF activity, protein serine/threonine kinase activity CC: nucleus, plasma membrane BP: protein amino acid phosphorylation | MA1281.1 | 20 | 1.3e-372 | 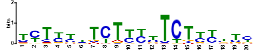 | Motif 1 |
| MF: TF activity, protein binding, protein serine/threonine kinase activity CC: nucleus   BP: regulation of transcription, DNA-dependent | MA1404.1 | 21 | 2.1e-025 | 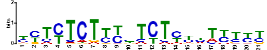 | Motif 2 |
|  | MA1160.1 | 20 | 1.4e-014 | 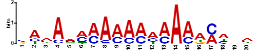 | Motif 3 |
| CC: nucleus, chloroplast thylakoid membrane, mitochondrion MF: TF activity, ATP binding | MA1231.1 | 38 | 2.9e+002 | 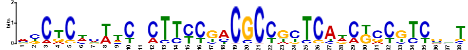 | Motif 4 |
| CC: mitochondrion, chloroplast | MA1319.1 | 41 | 1.1e+003 | 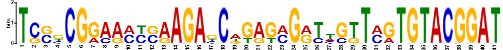 | Motif 5 |
| CC: mitochondrion, chloroplast | MA1353.1 | 49 | 5.6e+004 | 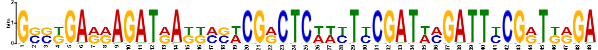 | Motif 6 |
|  | MA0573.1 | 43 | 1.0e+005 | 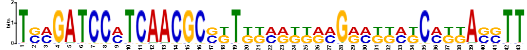 | Motif 7 |
| CC: mitochondrion, chloroplast MF: nucleotide binding, ATP-dependent helicase activity | MA0283.1 | 11 | 3.4e+006 | 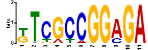 | Motif 8 |
| MF: structural constituent of ribosome BP: translation CC: cytosolic large/ small ribosomal subunit, nucleolus | MA1353.1 | 11 | 5.7e+006 | 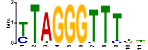 | Motif 9 |
| BP: protein amino acid phosphorylation | MA0944.1 | 29 | 8.1e+006 | 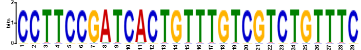 | Motif 10 |
|  | MA1141.1 | 28 | 1.6e+007 | 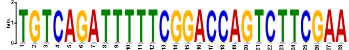 | Motif 11 |

**Table S10. c) The conserved cis-acting elements found in promoter of aphid DEGs by the MEME analysis.**

| **Significant GO term identified by GOMO** | **Best match in JASPAR and PLACE** | **Width** | ***E-value*** | **Motif Logo** | **Motif name** |
| --- | --- | --- | --- | --- | --- |
| MF: TF activity, protein serine/threonine kinase activity CC: nucleus, plasma membrane   BP: protein amino acid phosphorylation | MA1267.1 | 29 | 7.2e-038 | 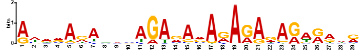 | Motif 1 |
| MF: TF activity CC: nucleus, plasma membrane BP: transmembrane receptor protein tyrosine kinase signaling pathway, protein amino acid phosphorylation | MA0543.1 | 20 | 1.1e-007 | 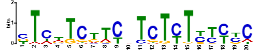 | Motif 2 |
| MF: TF activity CC: plasma membrane, nucleus BP: protein amino acid phosphorylation, regulation of transcription, DNA-dependent | MA0528.1 | 21 | 3.4e+002 | 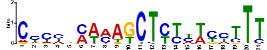 | Motif 3 |
| MF: TF activity, microtubule motor activity CC: chloroplast, nucleus BP: regulation of transcription, DNA-dependent | MA1402.1 | 21 | 7.6e+006 | 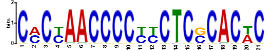 | Motif 4 |
| CC: mitochondrion, chloroplast stroma | MA0580.1 | 17 | 4.9e+006 | 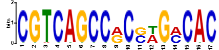 | Motif 5 |
| CC: chloroplast stroma, mitochondrion BP: DNA replication initiation, translation MF: structural constituent of ribosome | MA1242.1 | 15 | 1.0e+007 | 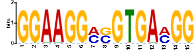 | Motif 6 |
| CC: chloroplast | MA0608.1 | 13 | 1.1e+007 | 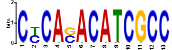 | Motif 7 |
|  | MA0652.1 | 21 | 1.7e+007 | 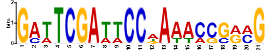 | Motif 8 |
| CC: mitochondrion, chloroplast envelope, chloroplast stroma BP: translation MF: structural constituent of ribosome | MA0283.1 | 21 | 1.8e+007 | 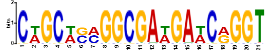 | Motif 9 |
| BP: phenylpropanoid biosynthetic process | MA1039.1 | 8 | 2.0e+007 | 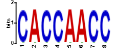 | Motif 10 |
|  | MA0611.1 | 28 | 2.1e+007 | 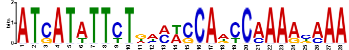 | Motif 11 |

**Table S10. d) The conserved cis-acting elements found in promoter of fungal-aphid DEGs by the MEME analysis.**

| **Significant GO term identified by GOMO** | **Best match in JASPAR and PLACE** | **Width** | ***E-value*** | **Motif Logo** | **Motif name** |
| --- | --- | --- | --- | --- | --- |
| MF: TF activity, protein serine/threonine kinase activity, protein binding CC: nucleus BP: regulation of transcription, DNA-dependent | MA1404.1 | 21 | 5.6e-312 | 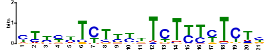 | Motif 1 |
| CC: chloroplast, plasma membrane, nucleus MF: RNA binding, ATP binding | MA1278.1 | 11 | 1.4e-027 | 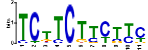 | Motif 2 |
| MF: structural constituent of ribosome BP: translation CC: cytosolic large/ small ribosomal subunit,  nucleolus | MA1353.1 | 11 | 9.9e-022 | 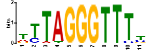 | Motif 3 |
| MF: TF activity, protein serine/threonine kinase activity, protein binding CC: nucleus BP: transmembrane receptor protein tyrosine kinase signaling pathway | MA1268.1 | 28 | 6.0e-004 | 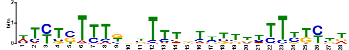 | Motif 4 |
| CC: mitochondrion, nucleus, chloroplast | MA1382.1 | 50 | 5.2e+002 | 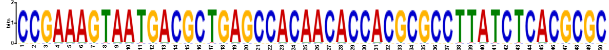 | Motif 5 |
| MF: TF activity CC: nucleus, plasma membrane BP: regulation of transcription, DNA-dependent, ovule development | MA1403.1 | 49 | 1.6e+004 | 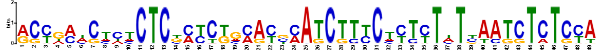 | Motif 6 |
|  | MA1215.1 | 46 | 2.4e+005 | 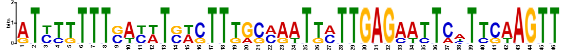 | Motif 7 |
| CC: mitochondrion, chloroplast stroma, chloroplast thylakoid membrane MF: RNA binding | MA1222.1 | 21 | 7.2e+006 | 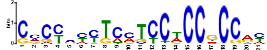 | Motif 8 |
| MF: TF activity, transcription activator activity CC: nucleus, plasma membrane BP: leaf development | MA1404.1 | 21 | 7.7e+006 | 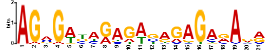 | Motif 9 |
| CC: chloroplast | MA0129.1 | 27 | 6.8e+007 | 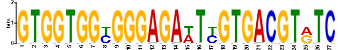 | Motif 10 |
| CC: chloroplast, mitochondrion | MA0456.1 | 16 | 1.4e+008 | 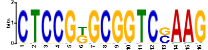 | Motif 11 |
