# Supplementary material for: Comparative meta-analysis of barely transcriptome: Pathogen type determines host preference
Source: PLoS One. 2025 Jun 30;20(6):e0320708. doi: 10.1371/journal.pone.0320708 (PMC12208424; doi:10.1371/journal.pone.0320708)
Supplement: Attachment — Submitted filename: Response to Reviewers.docx [file pone.0320708.s014.docx]

**Reviewer #1:** Summary

The authors present a meta-analysis and co-expression network analysis of barley transcriptomic responses to fungal infections, aphid infestations, and hormone treatments, using microarray data. The study highlights interesting hub genes, transcription factors, cis-regulatory elements, and miRNAs, along with associated GO terms. The authors suggest that their findings offer insights into barley's crosstalk between biotic stresses and introduce candidate genes for potential genetic engineering applications.

Major Points

1. The manuscript was provided without line numbers, making it difficult to refer to specific sections during the review. Please ensure line numbers are added in future submissions.

Answer: Line numbers were added in the revised version of the manuscript.

2. While the current analysis is valid for the datasets used, it would be ideal to incorporate RNA-seq data, which would provide higher resolution and greater dynamic range compared to microarrays. This could either be incorporated into the meta-analysis and subsequent analyses or could be used as validation for the conclusions made. If relevant RNA-seq data is not available for barley under similar conditions, this limitation should be acknowledged in the discussion.

Answer: We really appreciate you for your comment. Actually, we recognized some RNA-seq data, but they are not similar to our microarray conditions that we explained this issue based on your comment in the conclusion. We mentioned some detailed reasons here:

- We have four distinct microarray datasets (fungal, hormonal, pest, and fungal-pest interactions) and the lack of similar RNA-seq data makes the comparison impossible.

- We have analyzed rather big data and despite their limitations, the results remained statistically significant in different analyses. They have shown specific transcriptomic responses of barley to the target pathogens.

- In addition, the results demonstrate meaningful statistical relevance, supported by robust cross-validation methods detailed in the manuscript. This validation (LOOCV) approach ensures that our findings are reliable.

Despite our extensive validation analyses, we acknowledge that the lack of relevant RNA-seq data under similar conditions is a limitation. We have addressed these in the conclusion section to provide a comprehensive understanding of the limitations faced in our research.

3. Given that the study is based on a relatively small gene set (“approximately 2,000 genes”), the scope of biological conclusions drawn from the GO analysis may be limited. I suggest the authors clarify the limitations of their findings and consider expanding the study with additional data, if possible, or tempering the claims made based on this limited set.

Answer: Thank you for your valuable comment. In the manuscript, we have mistakenly stated, "Each dataset contains contained more than 16,300 probeset IDs representing approximately 2,000 genes." The correct sentence is: "Each dataset contains more than 20,000 probeset IDs representing approximately 7,000 genes." We corrected this accidentally mistake in the text. After normalization of the datasets, we did a series of analyses and filtering based on the P-value or FDR ≤ 0.05 mentioned in the text, and we identified a set of differentially expressed genes (DEGs) that are statistically significant. Finally, the GO analysis has shown statistically significant pathways specifically related to the target pathogens and molecular responses. We acknowledge that using a smaller gene set may limit some biological conclusions drawn from GO analysis. In addition, we have addressed the possible limitations in the manuscript based on your comment and emphasized that is better to do further research with additional datasets to enhance the result robustness.

4. Barley and Arabidopsis are evolutionarily distant, and while Arabidopsis serves as a useful model, there are significant differences in gene regulation and promoter architecture between the species. The use of Arabidopsis promoters to infer functionality in barley is problematic, as promoter elements can differ even within related plant clades. I recommend the authors acknowledge this as a significant limitation and be cautious when making strong functional inferences based on Arabidopsis orthologs and promoter sequences.

Answer: Thank you for your valuable feedback.

Actually, we have a big list of candidate genes used to promoter analysis. This impossible to analysis those individually. Therefore, we have to analysis these genes as a list. In addition, the practical databases like BioMart and GOMO show the best results based on the Arabidopsis and if we use the barley IDs as impute, we lost a lot of data. Of course, we have chosen the highly conserve regulatory motifs that are significantly specific relationships with target pathogens and molecular responses in barley. Although many authoritative papers have used the same approch, we have outlined possible limitations.

Anyway, we improved the text based on your comment and acknowledged the possible limitations in the text. We have addressed that functional roles of regulatory cis-acting elements derived from Arabidopsis may not accurately predict their roles in barley due to these evolutionary distinctions. Therefore, we recommend a cautious interpretation of results derived from these analyses in our manuscript. We hope this additional context will satisfy the reviewer's concerns. We aim to bridge the gap in knowledge and provide a foundation for future research that can validate these findings in the context of barley-specific biology.

In addition, some detailed reasons are here:

- Arabidopsis has a well-annotated genome with a vast array of functional data, which can provide insights into gene functions and regulatory mechanisms that may not yet be available for barley. This enables us to infer potential roles of barley genes based on their orthologs in Arabidopsis.

- Despite the evolutionary differences, certain promoter motifs and transcription factor binding sites are conserved across species. Research has demonstrated that many transcriptional regulatory mechanisms remain analogous, enabling meaningful insights into barley gene expression by examining their Arabidopsis counterparts.

5. The discussion section is overly long and frequently references other studies that are not directly related to the data presented in this manuscript. For instance, the section on GLVs (green leaf volatiles) introduces concepts that are not mentioned earlier in the paper, making it seem disconnected from the main results. Furthermore, claims such as "This meta-analysis confirmed that fungi induce more GLVs than insects" and "our overall meta-analysis shows that crops…result in notably elevated levels of phenolic compounds…" are not supported by the data, as the study does not quantify these compounds but only examines gene expression related to their production. Please revise the discussion to focus more on your study's findings.

Answer: Thank you, we removed unrelated explanations that did not align with the main objectives of the manuscript. Additionally, we have condensed and specified the discussion to focus more directly on our results.

6. I appreciate that the authors are non-native English speakers; however, certain sections, particularly the abstract and introduction, are difficult to read due to incorrect sentence structure, grammar, and punctuation. I recommend that the manuscript be reviewed by a native English speaker or a professional editor to improve clarity and readability.

Answer: Thank you, we appreciate your understanding that we are non-native English speakers. In response to your comments, we have thoroughly revised the manuscript, focusing specifically on the abstract and introduction. We have made significant improvements to the sentence structure, grammar, and punctuation to enhance clarity and readability.

Minor Points

- There are numerous typographical errors throughout the manuscript. However, without line numbers, it is difficult to provide specific examples. Please proofread carefully.

Answer: Thank you for your feedback. We have carefully proofread the manuscript and corrected all typographical and writing errors. Additionally, we have included line numbers in the revised version to facilitate easier reference for any future comments or suggestions.

- Figure 1 is overly complex and lacks clarity. For example, no explanation is provided to differentiate the black boxes from the purple ellipses. According to the methodology, DEGs from the meta-analysis and co-expression analysis should feed into subsequent analyses (lateral analysis, GO enrichment), but this is not shown in the figure. Moreover, it seems to suggest that only "Fungi" data is used for co-expression analysis, only "Insect" for network analysis, and both "Insect & Fungi" for cis-acting element analysis, which contradicts the text. I recommend reformatting this figure to make the workflow clearer and more legible.

Answer: Thank you for your exact comments. We replaced the figure with a simple and comprehensive flowchart to clear up the confusion. Additionally, I have ensured that the workflow accurately reflects the methodology, illustrating how the DEGs identified from meta-analysis and the modules derived from co-expression analysis feed into GO enrichment. PPI interaction network, and cross-validation were performed for the co-expression modules identified. We have also clearly indicated that DEGs obtained from the meta-analysis are utilized for lateral analyses, such as identifying transcription factors, protein kinases, microRNAs, and ultimately for cis-acting element analysis.

- Figure 2 is not directly referenced in the text, causing the subsequent figure numbering to be out of sync. Please ensure all figures are referenced in the text and check that the correct figure numbers are used. Also, I find it unclear how upregulated and downregulated DEGs can overlap in this figure.

Answer: Thank you for your exact comment. Figure 2 is one step of data processing and checking during the meta-analysis and before the filtering of DEGs, thus does not represent the final results. This figure is accidentally presented and no need to present as a main figure. In this Venn diagram we checked the possible overlap between the up-regulated and down-regulated DEGs and have selected each overlap up or down gene based on the lowest and most significant P-value, in this case there finally will be no overlap. We appreciate your understanding and will ensure that all figures are correctly referenced and numbered in the revised submission.

- The number of genes associated with each GO term in Figure 4 does not match the values mentioned in the text. For instance, "protein folding" shows fewer than 25 genes in the figure 4b, though 7% of 2,531 genes should yield around 177 genes. Similarly, "response to reactive oxygen species" shows 50+ genes in the figure 4c, but according to the text, 2% of 62 DEGs is approximately 1 gene. These inconsistencies need to be corrected for accuracy.

Answer: We appreciate your attention to the details and improving our manuscript, which is crucial for ensuring the accuracy of our findings. In our initial analysis, we calculated the percentages based on the total number of genes that were significantly enriched within each GO term. This approach may have led to confusion and inconsistencies in our reporting. In response to your comment, we have thoroughly reviewed our calculations and made the necessary corrections. Specifically, we recalculated the number of genes associated with each GO term based on the total number of DEGs identified in our meta-analysis. We have ensured that these figures now align with the percentages presented in the text. We have updated both Figure 4 and the corresponding sections of the manuscript to reflect these corrections.

- The "PRISMA 2020 checklist" supplementary file contains important information about dataset adherence to MIAME guidelines, which is not addressed in the main manuscript. This information should be incorporated into the manuscript, as it is relevant to the quality of the data used.

Answer: Thank you for your valuable feedback regarding the inclusion of information from the "PRISMA 2020 checklist" supplementary file. In response, we have added detailed explanations in the "Methods" section, specifically the subsection "Data collecting". This addition highlights how our dataset complies with MIAME guidelines and emphasizes its relevance to the quality of the data used in our study.

**Reviewer #2:**

1) I think that the choice of research content is more meaningful. However, there are a lot of errors in the content, and it seems that the authors submitted this manuscript without checking it carefully, for example, there is an intersection of the Venn diagrams of the up- and down-regulated genes, and Fig2 in the manuscript actually refers to Fig3.

Answer: Thank you for your valuable feedback on our manuscript. We have carefully reviewed and revised the manuscript to address the errors you pointed out and similar items. Specifically, we have corrected the references to figures and meticulously checked to ensure clarity and adherence to grammatical standards. We believe these revisions enhance the quality of our manuscript, and we hope that it now meets the standards expected for publication.

2) In addition, the discussion section, although it is written a lot, it is not focused, and does not discuss the results of their own research, it is more like a review.

Answer: We appreciate your insights and have made significant revisions to enhance the focus and relevance of this section. In our revised version, we have shortened the discussion and ensured that it directly addresses the results of our own research. Additionally, we have removed unrelated materials and sources to streamline the content and improve clarity.
