# Supplementary material for: Comparative meta-analysis of barely transcriptome: Pathogen type determines host preference
Source: PLoS One. 2025 Jun 30;20(6):e0320708. doi: 10.1371/journal.pone.0320708 (PMC12208424; doi:10.1371/journal.pone.0320708)
Supplement: Attachment — Submitted filename: Response_to_Reviewers_auresp_2.docx [file pone.0320708.s015.docx]

**Responses to the reviewers**

Reviewer #3: Comments

1- The experiment is interesting as explores genes involved in response of barley to pathogens attacks. As responses to biotic and abiotic stresses are sometime common in plant species, I recommend to read some interesting publications to address whether some of signaling and genes are common between the manuscript submitted and the following publications? Addressing such issues using the following citations may give better insight into better interpretation of the results in discussion or introduction parts of the manuscript.

Ravi S. et al. 2021. Development of an SNP Assay for Marker-Assisted Selection of Soil-Borne Rhizoctonia Solani AG-2-2-IIIB Resistance in Sugar Beet. Biology,11:49, //[doi.org/10.3390/biology11010049](http://doi.org/10.3390/biology11010049)

Shariatipur et al. 2021. Comparative Genomic Analysis of Quantitative Trait Loci Associated With Micronutrient Contents, Grain Quality, and Agronomic Traits in Wheat (Triticum aestivum L.). frontiers in Plant Science, //[doi.org/10.3389/fpls.2021.709817](http://doi.org/10.3389/fpls.2021.709817)

Shariatipur et al 2021. Meta-analysis of QTLome for grain zinc and iron contents in wheat (Triticum aestivum L.). Eyphytica 217, //[doi.org/10.1007/s10681-021-02818-8](http://doi.org/10.1007/s10681-021-02818-8)

Salami et al. 2023. Integration of genome wide association studies (GWAS), metabolomics and transcriptomics reveals phenolic acids and flavonoids associated genes and their regulatory elements under drought stress in rapeseed flowers. Frontiers in Plant Science, 14, 10.3389/fpls.2023.1249142

Salami et al. 2024. Dissection of quantitative trait nucleotides and candidate genes associated with agronomic and yield-related traits under drought stress in rapeseed varieties: integration of genome-wide association study and transcriptomic analysis. Frontiers in Plant Sciences, 15 [doi.org/10.3389/fpls.2024.1342359](http://doi.org/10.3389/fpls.2024.1342359)

Moazami et al. Optimization of agrobacterium mediated transformation of sugar beet: Glyphosate and insect pests resistance associated genes. Agronomy Journal, 112, //[doi.org/10.1002/agj2.20384](http://doi.org/10.1002/agj2.20384)

Answer: Thank you very much for your valuable suggestions about the references. We used these references and cited them in the introduction, results, and discussion.

2- In Abstract, add number of data samples used for this meta- analysis

Answer: Yes, we added.

3- Introduction is too long, I suggest to condense introduction and keep the most important sections related to the topic. I suggest merge some of similar paragraphs with same content

Answer: Thank you for your valuable feedback. We have merged similar paragraphs, removed redundant information, and focused on the most critical points to make it more concise and suitable. The structure is now more focused and avoids unnecessary repetition.

4- Add to the table: the references where the data in Table 1 retrieved

Answer: Yes, we added

5- Some of publications suggested above analyzed WGCNA (almost Salami et al. papers). I suggest to have a look on these papers for interpretation of similar results for discussion part of the paper.

Answer: Thank you very much. For sure, it was very useful. We used it and cited it.

6- Page 12, line 11, what does <> means? It is not needed as a start point in this line

Answer: We removed it.

7- Page 15 line 3, do not start a start sentence in a paragraphs with words such as, Next

Answer: Yes, we replaced them with more appropriate words.

Conclusion
8- conclusion is too long and boring for readers. Conclusion should be in 3-4 sentences showing the most important finding. Si, revise the conclusion and focus on 3-4 main outcomes.

Answer: We edited it based on your comment.

Reviewer #4:

General Comments:

Clarity and Conciseness:
9- The abstract contains technical terms and multiple ideas in each sentence, which may overwhelm the reader. Breaking down complex sentences into simpler ones would improve readability.

Answer: Yes, it was improved based on your comment.

Structure and Flow:
10- While the abstract covers objectives, methods, results, and implications, the transitions between sections are abrupt. Adding linking phrases to guide the reader through the narrative would enhance flow.

Answer: Yes, it was improved.

Focus:
11- The abstract appears to attempt to cover too much ground, from meta-analysis to gene network analysis and signaling pathways. Focusing on a few key findings and their significance would increase its impact.

Answer: Yes, it was improved.

Specific Comments:

Opening Sentence:
12- "Fungi and insects have contrasting effects that alter the preference and performance of pathogenesis in barley."
This statement is too general and lacks context. Specify the nature of these "contrasting effects" to provide clarity. For instance, do fungi promote pathogenesis while insects inhibit it?

Answer: We thank the reviewer for their comment. To address the concern, we have revised the opening sentence to specify and clarify the contrasting effects of fungi and insects on pathogenesis in barley.

Objective Statement:
13- "Therefore, the characterization of synergistic and antagonistic pathogen mechanisms is highly important to be explored."
Revise for conciseness and clarity. Suggested rewrite:
"Characterizing synergistic and antagonistic pathogen mechanisms is crucial for understanding pathogenesis in barley."

Answer: Thank you for your valuable feedback and constructive suggestion. We have revised the sentence as recommended to improve conciseness and clarity.

Methods Section:
14- "We performed meta-analysis and co-expression gene network analysis of the barley transcriptome in response to fungi, aphids, and hormones."
Include a brief mention of the dataset size or sources to strengthen this statement's credibility.

Answer: Yes, it was applied.

Results Section:
15- "The results showed that 1.1% of DEGs were common between fungal and aphid-related datasets and 0.1% of DEGs were shared among all datasets."

Clarify the significance of these percentages. For instance, why are these common DEGs important?

Answer: Thank you for your valuable feedback. We clarified these parts based on your comment.

16- "Promoter analysis revealed that AP2/EREBP and C2H2 zinc finger factors included the most frequent binding sites and played a more important role in connection with the SAR/ISR pathways."
Avoid vague terms like "more important role." Quantify or specify their role.

Answer: Thank you for your valuable feedback. We specified this part and other similar results.

Hub Genes:
17- "Gene network analysis allocated the DEGs into multiple modules with high co-expression and identified specific pathogen-responsive hub genes, including SSI2, PAD2, RPS1, RPS17, SHM1, CYP5, and RPL21C."
Consider separating these findings into a new sentence to avoid overloading the reader. Mention the biological relevance of these hub genes briefly.

Answer: Thank you for your valuable feedback. We improved the descriptions based on your comment.

Novel Genes:
18- "Moreover, novel hub genes with unknown functions were identified, including AT4G31530, AT5G54855, AT3G07640, and AT1G05720, which may influence host preference against biotrophic/hemibiotrophic pathogens and aphids."
Highlight the potential significance of these novel genes more explicitly. How might they be used in future research or applications?

Answer: Thank you for your valuable feedback. We clarified these parts based on your comment.

Conclusion:

19- "This is the first preliminary systems biology analysis of barley transcriptomic responses to heterotroph/biotroph cross-talk regarding the preference and performance of Rhopalosiphum padi, introducing valuable candidate genes that may be beneficial for accelerating genetic engineering programs."
This conclusion is lengthy and could be split into two sentences for clarity. Additionally, avoid terms like "preliminary" in an abstract unless necessary, as it may undermine the study's perceived value.

Answer: Thank you. We edited this sentence based on your comment.

Suggestions for Improvement:
20- Use active voice where possible to make the abstract more engaging.

Answer: Thank you for your comment. It was improved.

21- Add a sentence on the broader impact of this research, particularly for barley disease resistance or agricultural applications.

Answer: Yes, it was added.

22- Ensure all technical terms (e.g., DEGs, SAR, ISR) are either commonly understood in the field or briefly explained.

Answer: Thanks, we would like to clarify that all abbreviations terms, including DEGs (Differentially Expressed Genes), SAR (Systemic Acquired Resistance), and ISR (Induced Systemic Resistance), were fully defined upon their first use and abbreviations section. As suggested, we have added brief explanations for the terms SAR, ISR, and DEGs in the relevant paragraphs of the manuscript. These additions ensure that readers unfamiliar with these terms can easily understand their significance in the context of our study.

Reviewer #5: (No Response)

Reviewer #6:

23. Clarification of trophic levels in the introduction
In the third paragraph of the introduction, only secondary consumers are exemplified, while the other two levels are mentioned generally. It would be helpful to provide specific examples for primary and tertiary consumers to ensure a balanced comparison.

Answer: Thank you for your valuable comment. We have revised the introduction to include specific examples of primary/secondary/tertiary consumers to provide a more balanced and comprehensive discussion of trophic levels in the context of host-pathogen interactions.

24- Explanation of the variance threshold in meta-analysis
The manuscript states that 20% of DEGs with a variance of less than 0.2 were removed, but does not explain why 0.2 was chosen. The authors should justify this threshold.

Answer: Thank you for your valuable feedback. The choice of a variance threshold of 0.2 was based on statistical considerations to ensure the robustness and reliability of the DEGs identified in our study.

The MetaDE package, which implements the RankProd method, is designed to integrate multiple datasets and identify DEGs with consistent expression patterns across studies. The RankProd method is non-parametric and relies on rank-based statistics, making it robust to outliers and noise. Specifically, variance thresholds are commonly used to filter out genes with low variability, as these genes often contribute noise rather than meaningful biological signals. The increasing the variance > 0.2 led to the exclusion of key genes that have a lot of biological relevance. However, genes with very low variance (e.g., variance < 0.2) are often non-informative or exhibit minimal biological relevance, as they show little to no change in expression across conditions. Including such genes can increase the risk of false positives and unnecessary computational burden.

By removing the bottom 20% of DEGs with a variance of 0.2, we aimed to remove genes with low variety in different conditions or treatments and focus on genes with significant expression changes, resulting in improved statistical power and biological relevance of our meta-analysis. In meta-analysis, managing the large volume of data and reducing its complexity is very important. By eliminating genes with low variance, analyses become simpler and more understandable. This threshold was chosen based on empirical evidence from previous studies using similar approaches (for example, [Soltani et al. (2023), Ramasamy et al. (2008) and Hong et al. (2006]), where a variance cutoff of 0.2 effectively balanced the trade-off between retaining biologically relevant genes and reducing noise. We believe that using this threshold will increase the accuracy and validity of our meta-analysis results. We added a brief explanation to the Methods section of the manuscript to clarify this point.

25. Clarification of fold-change thresholds for up-regulated and down-regulated genes
The terms "up-regulated" and "down-regulated" are used, but no fold-change threshold is provided. The authors should specify criteria, such as "genes with a fold change of 2 or more are considered upregulated or downregulated."

Answer: Yes, we improved these results based on your comment.

26. Detailed description of DEG selection criteria
The manuscript lacks detailed information on the DEG selection process. The authors should clarify the criteria and statistical thresholds used.

Answer: We clarify that our selection criteria and statistical thresholds for DEGs included a log2 fold change (FC) greater than 1 or less than -1, coupled with a false discovery rate (FDR) of less than or equal to 0.05."
